# Incidence and mortality from adverse effects of medical treatment in the UK, 1990–2013: levels, trends, patterns and comparisons

**DOI:** 10.1093/intqhc/mzy068

**Published:** 2018-04-12

**Authors:** Raimundas Lunevicius, Juanita A Haagsma

**Affiliations:** 1 General Surgery Department, Aintree University Hospital NHS Foundation Trust, Lower Lane, Liverpool, UK; 2 University of Liverpool, School of Medicine, Liverpool, UK; 3 Erasmus MC, Erasmus University Medical Center, Rotterdam CA, The Netherlands; 4 Institute for Health Metrics and Evaluation, University of Washington, 2301 Fifth Avenue, Seattle, WA, USA

**Keywords:** adverse events, patient safety, patient outcomes (health status, quality of life, mortality), measurement of quality, mortality, complications, quality culture, quality management, health policy, healthcare system

## Abstract

**Objective:**

To present an update on incidence and mortality from adverse effects (AEs) of medical treatment in the UK, its four countries and nine English regions between 1990 and 2013.

**Design:**

Descriptive epidemiological study on AEs of medical treatment. AEs are shown as a single cause-of-injury category from the Global Burden of Disease (GBD) 2013 study.

**Data sources:**

The GBD 2013 interactive data visualisation tools ‘Epi Visualisation’ and ‘GBD Compare’.

**Outcome measures:**

The means of incidence and mortality rates with 95% uncertainty intervals (UIs). The estimates are age-standardised.

**Results:**

Incidence rate was 175 and 176 cases per 100 000 men, 173 and 174 cases per 100 000 women in 1990 and 2013, in the UK (UI 170–180). The mortality from AEs declined from 1.33 deaths (UI 0.99–1.5) to 0.92 deaths (UI 0.75–1.2) per 100 000 individuals in the UK between 1990 and 2013 (30.8% change). Although mortality trends were descending in every region of the UK, they varied by geography and gender. Mortality rates in Scotland, North East England and West Midlands were highest. Mortality rates in South England and Northern Ireland were lowest. In 2013, age-specific mortality rates were higher in males in all 20 age groups compared with females.

**Conclusions:**

Despite gains in reducing mortality from AEs of medical treatment in the UK between 1990 and 2013, the incidence of AEs remained the same. The results of this analysis suggest revising healthcare policies and programmes aimed to reduce incidence of AEs in the UK.

## Introduction

Comprehensive scans of research on harm related to adverse effects (AEs) arising from medical treatment in the UK show that knowledge on occurrence of AEs in the UK is based on the reviews of randomly drawn medical and nursing records from selected acute-care hospitals in UK at fixed time points [1, 2]. It is commonly stated that 8–12% of patients—or ~1 in 10 patients—admitted to a large acute-care hospital in UK experience an AE from medical treatment; a third of them usually lead to moderate or severe disability or death [3–5]. However, little is known about the levels, trends, and patterns in incidence and mortality rates from hospital setting AEs in the UK and its regions over time.

The Global Burden of Disease (GBD), injuries and risk factors study led by Murray [6, 7] provides a standardised approach to addressing this problem. Its interactive data visualisation system is a publicly available platform to retrieve, interpret and compare the standardised epidemiological estimates for AEs from medical treatment at the population level of individual countries and their regions by age, gender, geography and year [8, 9].

In this article built on the GBD 2013 study, we show the incidence and mortality of AEs resulting from medical treatment in the UK, its four constituent countries and nine regions of England [10] over a period of 24 years, 1990–2013. We show levels of, trends for and patterns from AEs resulting from medical treatment in the UK. The comparisons of AE incidence and death rates are provided for understanding the ranking of the UK on the scale of 33 highly industrialised high-income countries. It is important to note that AEs related to medical treatment in this article are defined as a single cause-of-injury category—an aggregate of 311 individual AEs resulting from medical treatment.

## Methods

### GBD study definition

The GBD study is a systematic, scientific enterprise to measure epidemiological levels of and quantify the magnitude of health loss from diseases, injuries and risk factors by age, gender and geography for specific points in time [6]. The GBD 2013 is a systematic, comprehensive effort to quantify health loss from 306 causes of diseases, 240 causes of death and 79 risk factors by gender and age groups between 1990 and 2013 for 188 countries. Detailed descriptions of the methodology of the GBD 2013 study are provided elsewhere [7, 11, 12].

### Adverse effects

An AE from medical treatment is a documented and coded injury resulting from a patient’s medical management. Only AEs of medical treatment at emergency departments and other hospital units were included into the process of estimations. A detailed list of the International Classification of Diseases, Ninth Revision, and the International Statistical Classification of Diseases and Related Health Problems, Tenth Revision, codes for AEs of medical treatment and other supplemental information on the burden of injury from AEs of medical treatment are available in the annex of Global Burden of Injury paper [12]. The terms ‘AEs from medical treatment’, ‘clinical AEs’, ‘adverse effects arising from medical treatment’ and ‘adverse effects resulting from medical treatment’ are synonymous in this paper.

### Data sources

For this analysis, we retrieved the age-standardised incidence and mortality means with 95% uncertainty intervals (UIs) for AEs of medical treatment from GBD 2013 interactive data visualisation tools ‘Epi Visualisation’ and ‘GBD Compare’ [8, 9]. The incidence and mortality estimates are age-standardised using the revised GBD 2013 standard population [7]. The UI expresses measurement error affecting data inputs. It is presented by the 2.5 and 97.5 centile values. Selection of the model—AEs of medical treatment—within the data visualisation tool allowed extraction of the pre-adjusted and post-adjusted AE incidence and mortality epidemiological estimates for every region of England, every constituent country of the UK, and every individual highly industrialised high-income country, by age, gender, geography and year. We used final fits for the years 1990, 1995, 2000, 2005, 2010 and 2013 for this study. Model results retrieved from both interactive data visualisation tools were entered into standard Microsoft® Excel Mac^2011^ spreadsheets. Subsequently, these results were further analysed using functions of this programme and bearing in mind the conception and aims of this study.

### Incidence

The incident case is defined as a newly diagnosed case with the AE resulting from medical treatment in a population in a given period—a year. Incidence expressed as a rate is defined as the number of new cases per person a year. It is approximately measured by taking the number of new cases in a year divided by the mid-year population of a country or region. Example: incidence at the level 0.0017558861581211 for one male individual in 2013 means that incidence of the particular condition is 176 new cases per 100 000 men in 2013. To estimate incidence rates with 95% UIs by age, gender, geography and year, a descriptive epidemiological meta-regression tool DisMod-MR 2.0 was applied. Information on estimated population size for the countries of the UK and the English regions in mid-1990 and mid-2013 was provided by Office for National Statistics, National Records of Scotland, Northern Ireland Statistics and Research Agency for the calculations of incident cases in men and women in 1990 and 2013 [13].

### Mortality

Mortality expressed as a rate is defined as the number of deaths due to AEs resulting from medical treatment per person a year among the entire population. For instance, the mortality rate from AEs from medical treatment 0.000012 for the UK’s men in 1990 means that there were 1.2 deaths per 100 000 or 12 deaths per 1 million men due to AEs resulting from medical treatment in the UK in 1990. To derive mortality rates with 95% UIs by age, gender, geography and year, the GBD cause of death ensemble modelling (CODEm) software was used.

### Percentage change and difference

The percentage change between two mean values represents the degree of change—percent increase, percent decrease or decline—at different points in time, i.e. between 1990 and 2013 in this paper. The percentage difference between two mean values was calculated to show a distribution of the differences between AE age-specific mortality rates in men and women of the UK in 2013, i.e. at the same time point.

### High-income countries

The set of 33 high-income countries includes four high-income Asia Pacific countries (Brunei, Japan, Singapore and South Korea), two Australasia countries (Australia and New Zealand), two North America countries (Canada and USA), three South American countries (Argentina, Chile and Uruguay) and 22 Western Europe countries (Andorra, Austria, Belgium, Cyprus, Denmark, Finland, France, Germany, Greece, Iceland, Ireland, Italy, Israel, Luxembourg, Malta, the Netherlands, Norway, Portugal, Spain, Sweden, Switzerland and the UK). The UK was compared with 32 other high-income countries to provide the meaningful comparisons of the rates of incidence and mortality from AEs of medical treatment.

## Results

### Incidence

**Figure 1:**
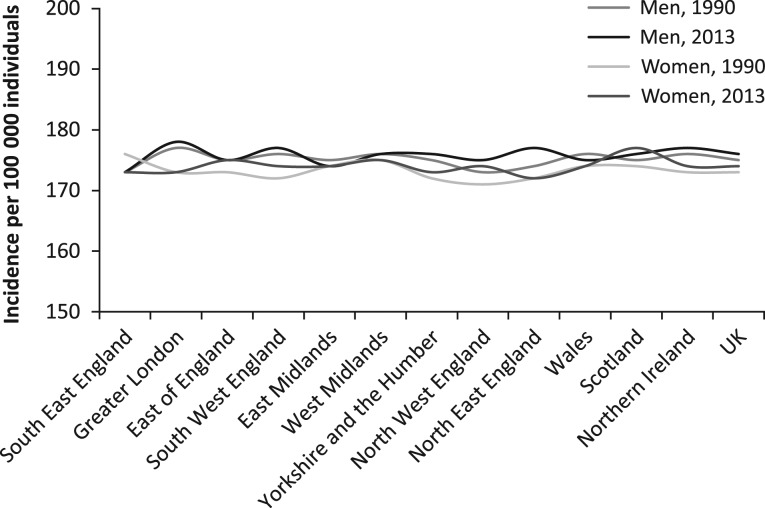
Trend line chart from the mean age-standardised incidence rates per 100 000 individuals with adverse effects resulting from medical treatment in the UK, by geography, gender and year.

Estimates of incident cases with 95% UIs from AEs arising from medical treatment in the UK in 1990 and 2013 is presented in Table 1. In 1990 and 2013, the age-standardised incidence rate was 175 and 176 per 100 000 men, 173 and 174 per 100 000 women in the UK (95% UI 170–180). Figure 1 demonstrates stability in incidence from clinical AEs among men and women in the geographical regions of the UK between 1990 and 2013.

**Table 1:** Incident cases with 95% uncertainty intervals of adverse effects resulting from medical treatment in the UK in 1990 and 2013, by gender and geographical region

	Men		Women	
	1990	2013	1990	2013
East of England	4363 (3993–4742)	5140 (4687–5566)	4489 (4148–4925)	5307 (4840–5747)
East Midlands	3422 (3136–3724)	3953 (3629–4309)	3538 (3253–3863)	4054 (3729–4428)
Greater London	5813 (5252–6236)	7401 (6665–7914)	6088 (5626–6681)	7346 (6802–8077)
North East	2183 (2004–2380)	2261 (2046–2429)	2293 (5641–6698)	2295 (2131–2531)
North West	5725 (5286–6278)	6119 (5592–6641)	6035 (5641–6698)	6285 (5773–6856)
South East	6404 (5925–7036)	7456 (6918–8215)	6839 (6232–7400)	7751 (7150–8491)
South West	3978 (3619–4297)	4674 (4225–5017)	4134 (3850–4572)	4766 (4380–5201)
West Midlands	4517 (4096–4864)	4926 (4486–5327)	4660 (4253–5051)	5030 (4593–5455)
Yorkshire and the Humber	4195 (3827–4545)	4633 (4208–4997)	4358 (4046–4805)	4685 (4332–5145)
Northern Ireland	1368 (1245–1478)	1590 (1435–1705)	1412 (1308–1554)	1627 (1492–1772)
Scotland	4279 (3910–4643)	4553 (4138–4914)	4585 (4220–5011)	4856 (4386–5208)
Wales	2442 (2215–2631)	2655 (2424–2879)	2573 (2363–2806)	2733 (2507–2978)
UK	48 690 (47 292–50 073)	55 368 (53 606–56 759)	51 006 (50 012–52 954)	56 671 (55 374–58 631)

**Figure 2:**
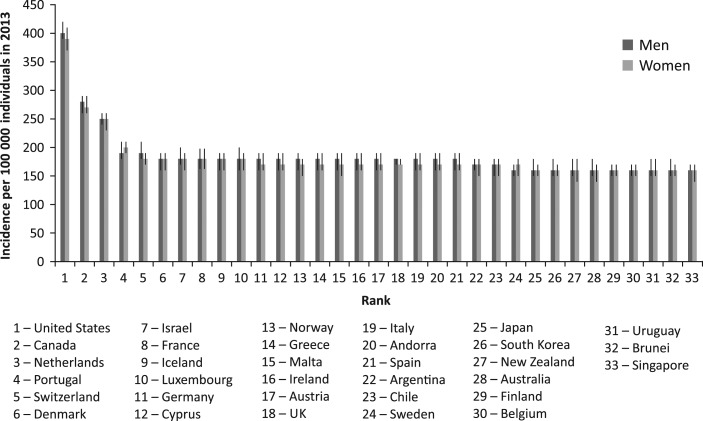
Similar age-standardised incidence rates with 95% uncertainty intervals for adverse effects resulting from medical treatment in 33 high-income countries in 2013, by gender.

Figure 2 shows age-standardised incidence rates with 95% UIs from 33 high-income countries in 2013, by gender. It illustrates a similar incidence rate among men and women in 30 countries out of 33. The USA, Canada and the Netherlands stand out with age-standardised incidence rates of 400, 280 and 250 cases per 100 000 men, and 390, 270 and 250 cases per 100 000 women in 2013, respectively. Comparison of age-standardised incidence rates of AE of 33 high-income countries over time shows that the age-standardised incidence rates of AEs resulting from medical treatment remained stable over time between 1990 and 2013 for both genders.

### Incidence by age and gender

**Figure 3:**
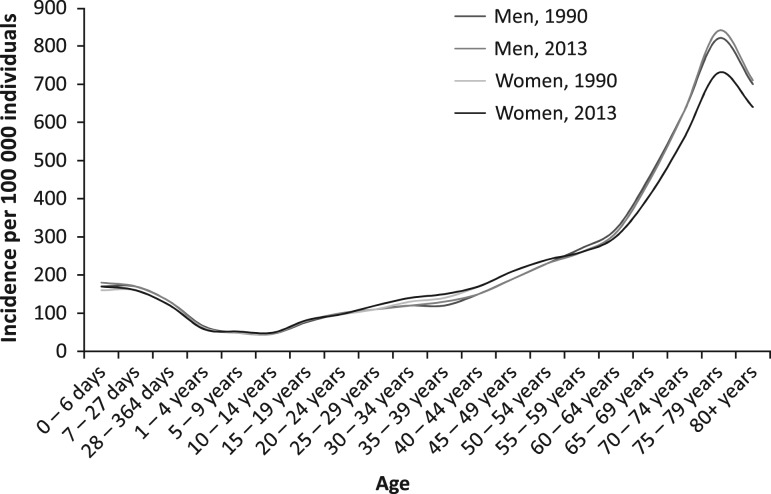
Trends from age-specific incidence rates for adverse effects resulting from medical treatment per 100 000 individuals in 1990 and 2013, by gender, UK.

Figure 3 shows the trends of age-specific incidence rates of AEs resulting from medical treatment in men and women in the UK in 1990 and 2013. All trend lines are gradually ascending from the age group 10–14. Trends are more distinct for men. The biggest difference in age-specific incidence rates among men and women observed is in the age group 75–79. The incidence rate in the 75–79 age groups was higher in men by 12.3% in 1990 and by 15.1% in 2013. The figure suggests a division of each trend line into four portions reflecting age groups 0–364 days (moderately high incidence rates), 1–9 years (lowest rates), 10–64 years (steadily increasing rates) and 65–80+ years (extremely high incidence rates). It reflects greater morbidity related to clinical AEs in neonates and elderly.

### Mortality

Table 2 provides information on a number of deaths and age-standardised mortality rates (ASMR) with 95% UIs from AEs resulting from medical treatment in individuals of both genders combined in 1990 and 2013, within the regional geography of England and a constituent country of the UK. The ASMR from AEs resulting from medical treatment declined from 1.33 deaths (95% UI 0.99–1.5) to 0.92 deaths (95% UI 0.75–1.2) per 100 000 individuals in the UK between 1990 and 2013 (30.8% change). The number of deaths decreased by 8.6% in the UK. The positive change in Greater London is notable—27.8% decrease in deaths and 37% change in ASMR from 1990 and 2013. In contrast, the number of deaths increased in Northern Ireland and Scotland by 13.5%. The decrease of ASMR in Scotland was the least within the geographical regions of the UK—13.7%.

**Table 2:** Deaths and age-standardised mortality rates (ASMR) with 95% UIs and percentage change from adverse effects resulting from medical treatment in men and women combined in the UK in 1990 and 2013, by regional geography

	Deaths in 1990	Deaths in 2013	Percentage change	ASMR per 100 000 in 1990	ASMR per 100 000 in 2013	Percentage change
East of England	93.33 (64.4–112.2)	90.95 (69.15–133.58)	−2.6	1.26 (0.88–1.5)	0.82 (0.63–1.17)	−34.9
East Midlands	74.93 (54.19–92.79)	71.74 (54.59–103.59)	−4.3	1.33 (0.96–1.62)	0.89 (0.69–1.25)	−33.1
Greater London	124.51 (87.64–147.68)	89.91 (65.53–127.27)	−27.8	1.35 (0.96–1.58)	0.85 (0.65–1.25)	−37.0
North East	55.49 (39.75–67.27)	48.75 (36.55–70.6)	−12.1	1.55 (1.12–1.88)	1.05 (0.8–1.49)	−32.3
North West	135.44 (92.27–162.13)	108.48 (82.56–151.96)	−19.9	1.38 (0.97–1.63)	0.9 (0.7–1.25)	−34.8
South East	127.41 (86.63–151.06)	116.08 (86.99–166.58)	−8.9	1.08 (0.76–1.27)	0.72 (0.54–1.01)	−33.3
South West	79.91 (54.13–96.07)	75.44 (57.52–104.17)	−5.6	1 (0.69–1.18)	0.68 (0.53–0.93)	−32.0
West Midlands	105.9 (75.16–128.16)	100.56 (75.99–136.71)	−5.0	1.49 (1.07–1.8)	1.03 (0.79–1.41)	−30.9
Yorkshire and the Humber	93.78 (64.17–112.18)	77.47 (60.92–116.4)	−17.4	1.32 (0.92–1.56)	0.86 (0.68–1.26)	−34.9
England	890.7 (644.41–999.76)	776.39 (645.07–1045.15)	−12.7	1.28 (0.94–1.44)	0.85 (0.7–1.14)	−33.6
Northern Ireland	15.92 (12.31–20.04)	18.4 (14.18–24.46)	13.5	0.84 (0.65–1.05)	0.68 (0.53–0.91)	−19.1
Scotland	137.94 (108.35–174.1)	159.49 (105.54–194.22)	13.5	1.99 (1.56–2.48)	1.73 (1.18–2.1)	−13.7
Wales	53.61 (37.64–64.25)	49.46 (37.28–69.95)	−7.7	1.24 (0.88–1.47)	0.87 (0.68–1.19)	−29.8
UK	1098.17 (805.99–1236.6)	1003.75 (815.75–1302.58)	−8.6	1.33 (0.99–1.5)	0.92 (0.75–1.2)	−30.8

UI, uncertainty interval (estimates in brackets); ASMR, age-standardised mortality rate per 100 000 individuals. Estimates for years 1990, 1995, 2000, 2005, 2010 and 2013 are available at http://vizhub.healthdata.org/gbd-compare.

**Figure 4:**
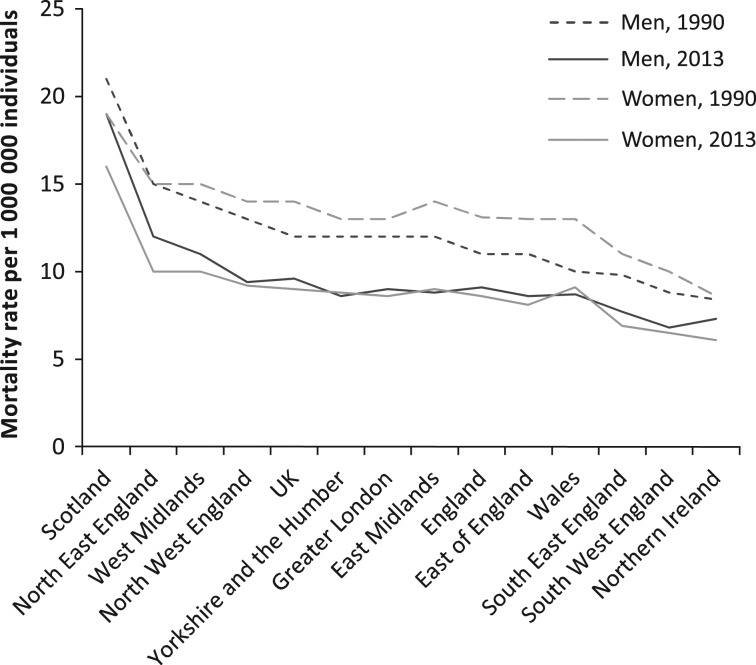
Variations in age-standardised mortality rates per 1 000 000 individuals from adverse effects resulting from medical treatment, by regional geography of the UK, gender and year; data sorted in descending order for men for the year 1990.

As shown in Fig. 4, ASMR trends were descending in every region of the UK from 1990 to 2013. However, the ASMRs varied by geography and gender. ASMRs in Scotland, North East England and West Midlands were highest, whereas ASMRs in Northern Ireland and both regions of South England were lowest. Overall, progress in all regions of England and Wales was achieved.

**Figure 5:**
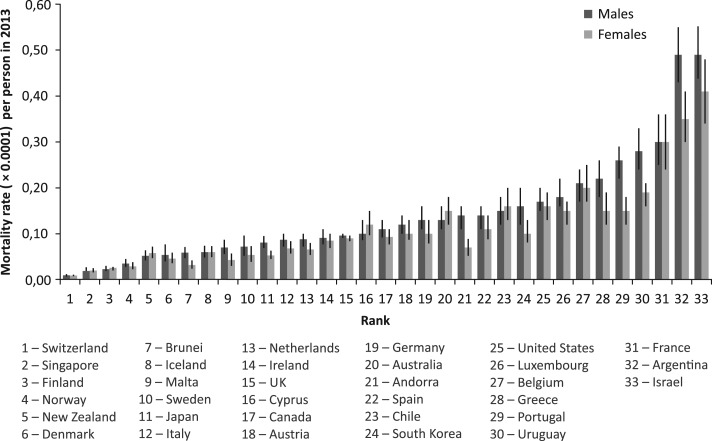
Significant variations in mortality rates from adverse effects resulting from medical treatment with 95% uncertainty intervals per person in 2013, by high-income country and gender; estimates sorted by ascending mortality rates in men.

Figure 5 shows notable variations in ASMRs from AEs of medical treatment in 2013, by high-income country. Comparison of ASMRs among women showed that the UK was performing worse than Switzerland by 9.9 times, Singapore by 4.5 times, Finland by 3.5 times, Norway by 3.1 times, Brunei by 2.8 times and Malta by 2.1 times in 2013. In men, the UK was performing worse than Switzerland by 10.1 times, Singapore by 5.1 times, Finland by 4.2 times and Norway by 2.7 times.

### Mortality by age and gender

**Figure 6:**
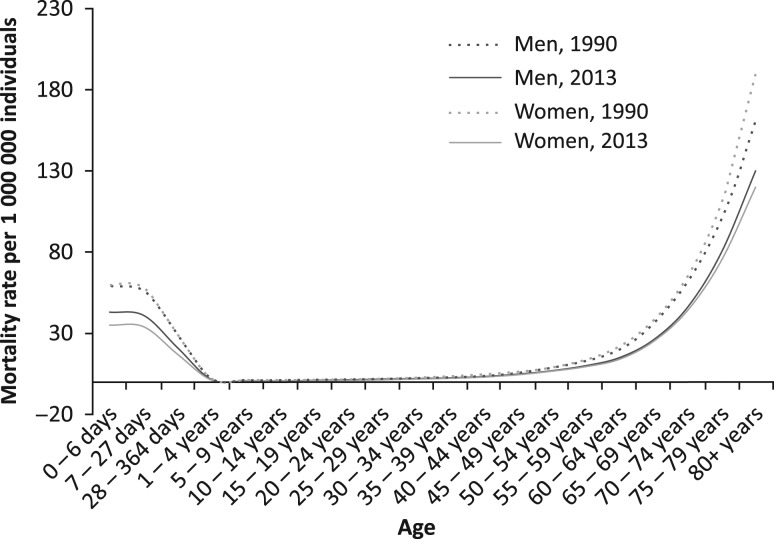
Pattern of age-specific mortality rates from adverse effects resulting from medical treatment, by gender and year, UK.

Figure 6 depicts a pattern of age-specific mortality rates from AEs resulting from medical treatment among men and women in the UK. It shows that age-specific mortality rates are comparable in newborn infants and in 70–74-year-old individuals. All four trend lines for age-specific mortality begin to markedly increase in the age group of 60–64 years. The highest age-specific mortality rates were in the age group ≥80 with 130 deaths per 1 million men and 120 deaths per 1 million women in 2013.

**Figure 7:**
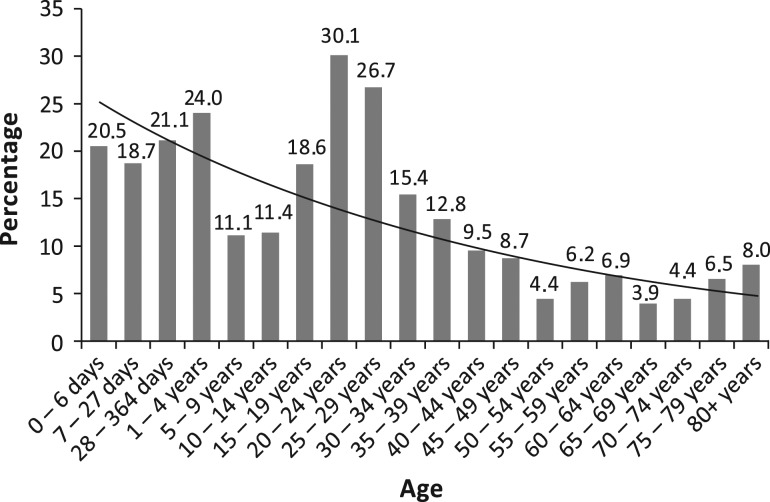
Distribution of percentage differences between adverse effect age-specific mortality rates in men and women with exponential trend line, UK, 2013; mortality rates are higher in all age groups in men.

In 1990, nearly all age-specific mortality rates—age groups from 10 to 24 years were the exceptions—were higher among women. In contrast, they were universally higher among men in all 20 age groups in 2013 in the UK. Figure 7 highlights the distribution of percentage differences between AE age-specific mortality rates in men and women in the UK in 2013. The greatest difference in age-specific mortality rates was observed in the age groups 20–24 and 25–29, 30.1% and 26.7%, respectively. The descending trend line shows that a factor of male gender for mortality from AEs arising from medical treatment is diminishing in the population of older individuals.

## Discussion

The key epidemiological estimates for AEs of medical treatment across age, gender, time and place have recently been publicised at the global level [7, 11, 14]. In the current study, we aimed to provide a description of levels, trends, and patterns of incidence and mortality from AEs of medical treatment in the countries of the UK and the regions of England from 1990 to 2013. The comparisons of AE incidence and death rates for the high-income countries are provided in this study to pinpoint ranking of the UK on the scale of 33 industrialised countries.

This paper shows that reduction in incidence and mortality from AEs resulting from medical treatment is a challenging task for every high-income country. The decline in ASMR from 1990 to 2013 indicates the progress in the UK in improving clinical outcomes related to AEs arising from medical treatment. However, age-standardised incidence rates did not change markedly in the UK between 1990 and 2013. They varied only minimally by region and gender. Interestingly, the levels of age-standardised incidence rates were similar in the majority of high-income countries.

Despite the reduction of ASMR from AEs resulting from medical treatment by 30.8% in the UK from 1990 and 2013, ASMRs varied visibly by the regional geography of the state. It is important to recognise that disparities persisted between North England and South England, Scotland and England, East Midlands and West Midlands. This analysis shows the lowest ASMRs in Northern Ireland and the highest ASMRs in Scotland. This phenomenon, therefore, has to be further discussed in editorials and national forums [15], for inequalities in mortality rates in the regions of the UK—where healthcare service is universal, comprehensive and equitable, and where there is nearly identical incidence—should not be tolerated. It is very likely that it may be related to a not-proportionate healthcare spending within the UK [16].

Comparison of age-specific mortality rates by gender revealed higher rates in all 20 age groups of men in 2013, with the highest age-specific mortality rates in newborn infants and 70–74-year-old men and women. This indicates that gender may be an important factor in mortality from clinical AEs.

The number of deaths from AEs resulting from medical treatment was stagnant in the UK between 1990 and 2013, ~1000 a year, as declining ASMRs did not outpace the increase in population of the UK. Such patterns suggest that death levels will not change in the UK if additional or entirely new strategies and measures for prevention of clinical AEs in the UK are not designed and implemented.

Besides the strengths of this paper, a few limitations have to be mentioned. First, the actual age-standardised incidence and mortality due to AEs resulting from medical treatment can differ from the GBD 2013 study estimates as culture of under-reporting and not-coding of clinical AEs prevails in healthcare facilities of the UK [17, 18]. Furthermore, the correct estimation of mortality from AEs resulting from medical treatment is even more hindered by the unavoidable overlap of the natural cause of the underlying illness itself and a clinical AE. Second, the definition of AEs resulting from medical treatment and the selection of codes for these could be questioned and challenged, as more ICD-10 codes may have been taken into account when estimating the burden of AEs resulting from medical treatment. The third limitation of this study is related to a bias occurring from mismanagement of the patients due to problems related to inadequate infrastructure resources, medical understaffing and ineffective communication that may often result in delays of timely treatment [19, 20]. Such clinical events are regarded as clinical incidents, for they do have clinical consequences to the patients and to the entire medical care system [21]. However, they are usually not coded as AEs resulting from medical treatment and, therefore, not reported to national registration systems in the form of ICD codes.

This review may have several educational and practical implications. As progress has not been made to achieve a reduction in the age-standardised incidence rate of AEs resulting from medical treatment across the UK between 1990 and 2013, it challenges the efficacy and effectiveness of the clinical risk management programmes and tools aimed to improve inpatient and outpatient safety and reduce the incidence of AEs resulting from medical treatment [22]. Therefore, new or newly revisited interventions to improve the process of mandatory, systematic and daily registration and coding of all AEs resulting from medical treatment would be beneficial for the healthcare system. Without precise daily capture and registration of clinical AEs of all categories of severity, and without regular epidemiological analysis and assessment of clinical AEs, a reduction of incidence and, subsequently, the burden of AEs resulting from medical treatment may be hardly achievable. Furthermore, as Buist and Middleton have recently suggested, entirely new concepts to better understand and manage more complex hospital setting AEs with the aim of designing interventions that are more focused on a hospital’s core business are needed [23, 24].

This study also implies that additional efforts are necessary to make the medical interventions safer for two marginal age groups of individuals—neonatal and elderly—in order to meet the demand generated by the growing number of the populations from these age groups in the UK [25, 26]. Finally, comparisons of ASMR within the set of 33 high-income countries and the modest (the 15^th^) ranking of the UK suggest that it is time to reconsider key points concerning the interaction between the patient and the healthcare professional [24].

## Conclusions

Progress has not been achieved in the reduction of incidence of AEs arising from medical care in the UK. AE incidence rate was essentially stable between 1990 and 2013. Age-specific incidence rates were highest in neonates and elderly. Although mortality rate trends are descending in every region of the UK, they varied by the regional geography, gender and age group. Mortality rates were higher among UK’s men in all 20 age groups in 2013. The UK is performing worse than Switzerland by ten times regarding mortality rates. Although synthesis of accurate identification, immediate electronic registration, meticulous coding of an AE resulting from medical treatment and regular analysis is a useful tool to learn lessons and reduce the occurrence and burden from clinical AEs, new strategies and policies that put the patient first are needed. In essence, it is all about the new shift in quality culture in healthcare organisation and provision in the UK.
